# Chimeric virus-like particles (VLPs) designed from shrimp nodavirus (MrNV) capsid protein specifically target EGFR-positive human colorectal cancer cells

**DOI:** 10.1038/s41598-021-95891-x

**Published:** 2021-08-16

**Authors:** Khwanthana Grataitong, Sébastien Huault, Charoonroj Chotwiwatthanakun, Pitchanee Jariyapong, Orawan Thongsum, Chidchanok Chawiwithaya, Krittalak Chakrabandhu, Anne-Odile Hueber, Wattana Weerachatyanukul

**Affiliations:** 1 Department of Anatomy, Faculty of Science, Mahidol University, Rama 6 Road, Bangkok, 10400 Thailand; 2 Department of Basic Medical Science, Faculty of Medicine Vajira Hospital, Navamindradhiraj University, Dusit, Bangkok, Thailand; 3 Center of Excellence for Shrimp Molecular Biology and Biotechnology, Faculty of Science, Mahidol University, Rama VI Road, Ratchathewi, Bangkok, Thailand; 4 Mahidol University, Nakhon Sawan Campus, Nakhon Sawan, Thailand; 5 Research Center of Excellence On Shrimp, Walailak University, Thasala District, Nakhon Si Thammarat, 80161 Thailand; 6 Université Côte d’Azur, CNRS, Inserm, Institut de Biologie Valrose (iBV), Nice, France

**Keywords:** Cancer, Nanoscience and technology

## Abstract

Recombinant MrNV capsid protein has been shown to effectively deliver plasmid DNA and dsRNA into Sf9 insect cells and shrimp tissues. To extend its application to cancer cell-targeting drug delivery, we created three different types of chimeric MrNV virus-like particles (VLPs) (R-MrNV, I-MrNV, and E-MrNV) that have specificity toward the epidermal growth factor receptor (EGFR), a cancer cell biomarker, by incorporating the EGFR-specific GE11 peptide at 3 different locations within the host cell recognition site of the capsid. All three chimeric MrNV-VLPs preserved the ability to form a mulberry-like VLP structure and to encapsulate EGFP DNA plasmid with an efficiency comparable to that previously reported for normal MrNV (N-MrNV). Compared to N-MrNV, the chimeric R-MrNV and E-MrNV carrying the exposed GE-11 peptide showed a significantly enhanced binding and internalization abilities that were specific towards EGFR expression in colorectal cancer cells (SW480). Specific targeting of chimeric MrNV to EGFR was proven by both EGFR silencing with siRNA vector and a competition with excess GE-11 peptide as well as the use of EGFR-negative colorectal cells (SW620) and breast cancer cells (MCF7). We demonstrated here that both chimeric R-MrNV and E-MrNV could be used to encapsulate cargo such as exogenous DNA and deliver it specifically to EGFR-positive cells. Our study presents the potential use of surface-modified VLPs of shrimp virus origin as nanocontainers for targeted cancer drug delivery.

## Introduction

Among several delivery systems at the nanoscale, virus-like particles (VLPs) designed from capsid proteins of many non-enveloped viruses are considered the most outstanding biomaterials that that surpass other biological containers^1^. This is simply due to their excellent physical properties such as self-assembling to form nano-sized symmetrical particles, controllable disassembly/reassembly, and practical surface functionality modifications through either chemical modification or genetic reengineering^2^. These surface modified VLPs, so-called “chimeric VLPs”, are intended for multiple research purposes. Interior modification mostly aims at improving both capabilities of VLPs to encapsulate their cargoes more effectively as well as traceability of these particles inside the targeted cells. On the other hand, exterior modification is aimed for either triggering immune response of the host cells or enhancing specific recognition of VLPs towards the desirable targeted cells or tissues^3^. The latter has been extensively reported with the objective to specifically deliver therapeutic compounds into the targeted cancer cells^4^. To date, several functional ligands or molecules that are known for their specific binding to cancer cells, e.g., antibody fragments^5^, short peptides^6^, DNA/RNA aptamers^7^ have been used to generate chimeric VLPs. Likewise, several kinds of VLPs have been used in the clinical trial as the hosted VLPs with their own intrinsic physical benefits including HBVc^8–10^, MS2^6,11^, Qβ^12,13^, and CPMV^14^.

*Macrobrachium rosenbergii* nodavirus (MrNV) is a shrimp infectious, non-enveloped virus with an icosahedral symmetry (T = 3) and a size of 26–27 nm in diameter^15–20^. Interestingly, only the RNA2 gene (out of several viral genes) is responsible for encoding capsid protein which makes it rather straightforward to produce recombinant protein to generate the self-assembling VLP^16,21^. Apart from this simplicity, its ability to withstand harsh conditions, particularly, strong digestive enzyme digestion as well as its controllable particle assembly has brought it an excellent deliverable nanocontainer. Principally, MrNV-VLPs could^22^. The controllability of MrNV-VLPs to encapsulate nucleotide-based agents (DNA vector and dsRNA) against the severely infectious virus through the uses of EGTA + DTT (specific calcium chelator and reducing agent, respectively) is also well established both in vitro and in vivo^22,23^.

**Figure 1:**
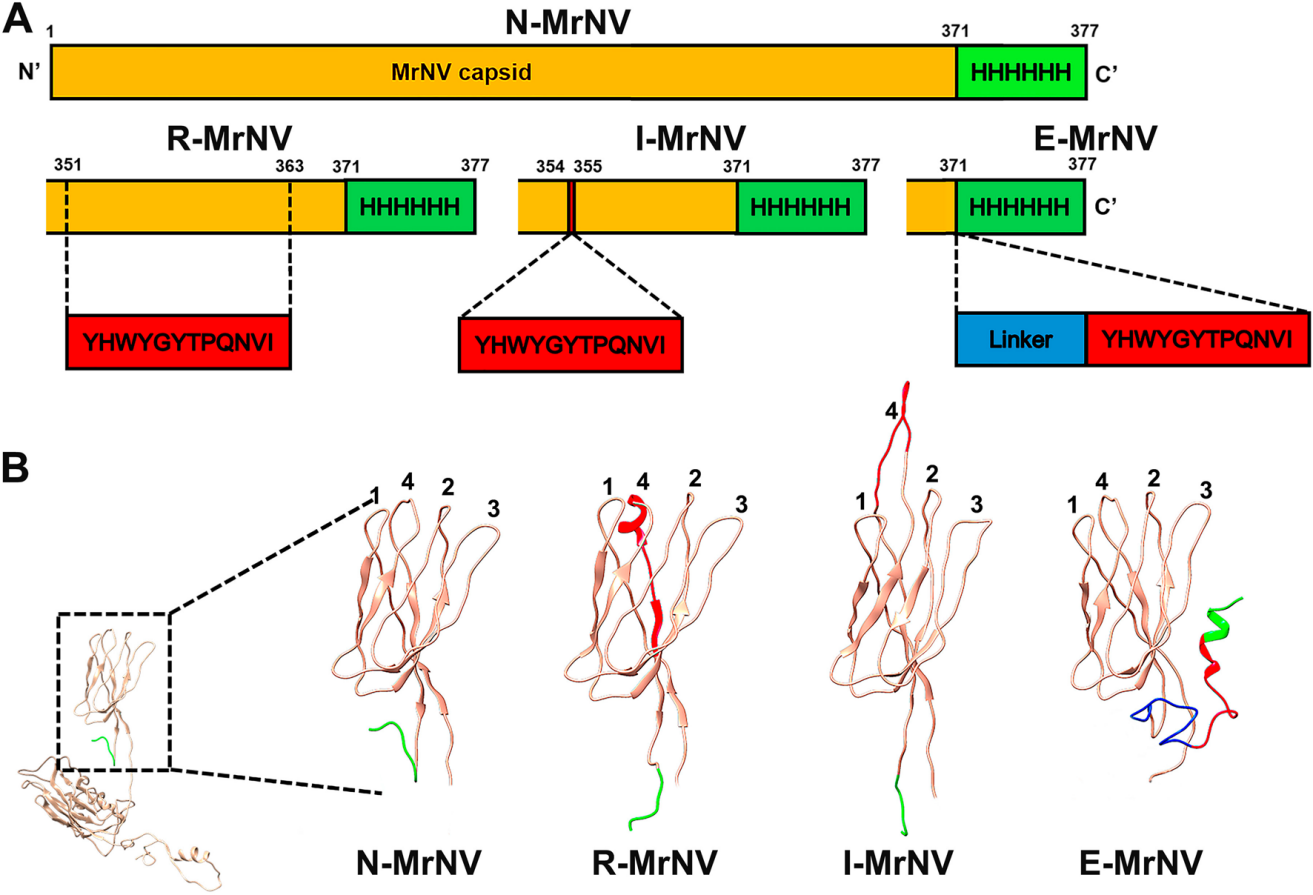
The schematic diagram of the construction of chimeric GE11 + MrNV-VLPs. In silico ribbon diagram of P-domain of 4 typed chimeric MrNV-VLPs including N-MrNV, R-MrNV, I-MrNV, and E-MrNV generated by Phyre2 algorithm version 2.0 (http://www.sbg.bio.ic.ac.uk/phyre2) and Chimera software version 1.14 (https://www.cgl.ucsf.edu/chimera). GE11 (red), linker (GGGGS)_3_ (blue) and 6 His-tag (green).

MrNV capsid consists of shell (S) domain and protruding (P) domain, the latter of which is the host cell recognition site^24^ (Fig. 1). It has been shown recently that the exterior modification of MrNV-VLP by adding the matrix 2 protein, a foreign epitope of influenza A (M2e) to its C-terminus induced type-1 T helper immune response in mice^25^. Similarly, adding chimeric hepatitis B surface antigen (HBsAg) to the MrNV-VLPs significantly increases the levels of natural killer and cytotoxic T cells as well as enhances interferon-gamma (IFN-γ) secretion in mice^26^. Notably, most of the placement of foreign epitopes into the VLP exterior is at any individual 4 loops of the protruding (P) domain (Fig. 1) located between amino acids 268–275 (loop 1), 296–303 (loop 2), 322–326 (loop 3), and 350–355 (loop 4)^24^. In this study, we explored the promising application of the physically stable MrNV VLP as nanocontainers for cancer cell-targeted drug delivery. We constructed 3 types of MrNV VLP bearing chimeric GE11 peptide which have been shown to target the epidermal growth factor receptor (EGFR) in several human cancer cells^1^. The resulting chimeric EGFR-targeting MrNV VLPs that we created exhibited great potential as nano-containers for targeted delivery of therapeutic compounds to cancer cells.

## Materials and methods

### Designing of chimeric GE11-MrNV-VLP variants

We designed chimeric GE11-MrNV-VLPs using the sequence information from normal MrNV (N-MrNV) capsid protein (Genbank accession No. EU150129) and the 12 amino-acid sequences of GE11 peptide, YHWYGYTPQNVI^27^. The GE11 peptide was placed at 3 different sites in the MrNV protruding (P) domain, creating 3 forms of chimeric VLPs (Fig. 1A). In the R-MrNV, 12 amino acids (residues 349–360) in the P-domain of MrNV were replaced with GE11 peptide. In the I-MrNV, the GE11 peptide was inserted between amino acids 354 and 355 of the P-domain. In the E-MrNV, a linker of 20 alanine residues followed by the GE11 peptide was added at the C-terminus of the MrNV capsids. Three dimensional (3D) homology-based models of all chimeric GE11-MrNV-VLPs were created by the Protein Homology/analogy and Recognition Engine version 2.0 (Phyre^2^) protein folding prediction server^28^. The MrNV template (PDB#6H2B) was chosen based on the scores obtained from fold recognition and threading algorithms in Phyre^2^. The icosahedral models of the chimeric MrNV-GE11-VLPs were created based on the icosahedral structure of N-MrNV as a referencing atomic structure displaying the highest secondary structure agreement with T = 3 quasi-equivalent^29^. All visualization was performed using the UCSF Chimera software (http://www.cgl.ucsf.edu).

### Expression and purification of chimeric GE11-MrNV-VLP

All of the chimeric MrNV-GE11 capsid sequences were synthesized and ligated into the pET16b expression vector (General Biosystems, Durham, NC). Transformation of the recombinant vector into competent *Escherichia coli* (BL21) was performed by the heat shock method (42 °C, 45 s) and immediately followed by ice incubation (5 min). Transformed *E*.* coli* were inoculated in SOC (Super Optimal Broth) medium (Invitrogen, Grand Island, NY), selected on the LB agar plates containing ampicillin (50 g/ml). The positive clones, verified by restriction endonuclease digestion and DNA sequencing, were transformed into *E. coli* and further inoculated in LB broth containing 50 µg/ml of ampicillin overnight at 37 °C until the absorbance of 0.6–0.8 at 600 nm (A600) was reached. Protein expression was induced by adding 1 mM IPTG to the culture followed by an overnight incubation (16 h, 25 °C). After induction, the cells were pelleted by centrifugation (5400×*g*, 10 min, 4 °C) and resuspended in PBS containing 500 mM NaCl and 2 mM phenylmethylsulfonyl fluoride (PMSF). The cells were ruptured by sonication (100 Hz, 20 s, 10 cycles) and centrifuged (12,000×*g*, 10 min). The collected supernatant was loaded onto a Nickel column chromatography. After several washes with a washing buffer (PBS, 500 mM NaCl, 20 mM imidazole, pH 7.4), the bound proteins were eluted with elution buffer (PBS, 500 mM NaCl, 250 mM imidazole, pH 7.4). The proteins were dialyzed against PBS overnight at 4 °C and further subjected to a sucrose gradient (10–40%) ultracentrifugation (245,000×*g*, 5 h, 4 °C). Each fraction was collected and analyzed by SDS-PAGE and immunoblotting. The concentration of purified GE11-MrNV-VLP was measured by the NanoDrop2000 Spectrophotometer (Thermo Fisher Scientific, Delaware, CA).

### Immunoblotting

Recombinant chimeric GE11-MrNV-VLPs obtained from all purification steps were resolved in 12.5% SDS-PAGE and Coomassie blue staining. The proteins were either transferred to a polyvinylidene fluoride (PVDF) membrane (Millipore Co., Billerica, MA) or directly blotted onto the membrane using a slot blot device They were .submerged in 2% BSA and 5% skim milk to block non-specific antibody staining and washed with PBS containing 0.05% Tween (PBST). Thereafter, transferred proteins were probed with either monoclonal anti-MrNV or anti-His or anti-EGFR or anti-GAPDH antibody at the dilution of 1:2000 (2 h, room temperature) followed by a goat anti-mouse IgG conjugated with horseradish peroxidase (HRP) at a dilution of 1:5000. After extensive washing with 0.05% PBST, the reactivity of antibody-antigen was detected by an enhanced chemiluminescence method using an ECL kit (Amersham Biosciences, Piscataway, NJ).

### Transmission electron microscopy

For VLP structure verification by transmission electron microscopy (TEM), 10 µl of purified chimeric MrNV-VLP was pipetted onto the carbon-coated EM grids (Electron Microscopy Sciences, Hatfield, PA) and allowed to stand for 30 s. Non-adhering VLPs were washed away by droplets of Milli-Q water and blotted away by a filtered paper. The adhered VLPs were stained with 2% uranyl acetate for 30 s and immediately blotted away by a filtered paper. Thereafter, the samples were viewed under a JEOL1230 transmission electron microscope operated at 120 kV (JEOL, Tokyo, Japan).

### Cell culture

Colorectal cancer cell lines, SW480 (EGFR-positive) and SW620 (EGFR-negative) were maintained in RPMI 1640 + Glutamax I medium supplemented with 10% fetal bovine serum (FBS). The breast cancer cell line, MCF-7 (EGFR-negative) were maintained in DMEM medium supplemented with 10% FBS.

### Binding and internalization of chimeric VLPs in SW480 cells

Cells at ~ 90% confluence were detached and seeded in 6-well plates (2 × 10^5^ cells/well) and cultured for 24 h before the binding and internalization assays. For binding experiments, the cells were pre-chilled at 4 °C and then incubated either with N-MrNV-VLP (control) or with chimeric GE11-MrNV-VLPs at the final concentration of 50 μg/ml or approximately 4.6 × 10^5^ VLP particles/ml at 4 °C for 2 h with gentle shaking. The excess or loosely bound VLPs were then washed from the cells by ice-cold culture media.

To test the specificity of EGFR-mediated VLP binding by siRNA silencing of EGFR SW480 cells were transfected with either EGFR siRNA (5′-AAGCTCACGCAGTTGGGCACT-3′) or control siRNA (Luciferase, 5′-CGUACGCGGAAUACUUCGA-3′) (Qiagen, Hilden, Germany) using a lipofectamine RNAiMAX reagent (Invitrogen, Eugene, CA) according to the manufacturer’s protocol for reverse transfection. Two days after siRNA transfection, cells were subjected to the binding assay as described above.

To verify the EGFR-specific binding of the GE11-MrNV VLPs by GE11 peptide competition assay, an excess amount of GE11 peptide or a scrambled peptide (control with the same amino acid composition, WQTNYIHPYVYG) was included in the cell culture medium (100 μg/ml) before the incubation of the cells with chimeric VLPs. Following the preincubation with free competing peptide, the cells were subjected to the VLP binding assay as described above. Subsequently, the cells were fixed with 4% paraformaldehyde, washed in PBS, and further processed for analyses by indirect immunofluorescence (IIF) or flow cytometry.

For VLP synchronized internalization test, following the VLP binding at 4 °C (without or with peptide competition) as described above, the VLP-containing medium was replaced with a new culture medium without VLPs at 4 °C. The cells were then incubated at 37 °C for 30 min in an incubator and further processed for IIF or flow cytometry as above.

### Delivery of encapsulated DNA cargo to colorectal cancer cells by VLPs

The encapsulation of pEGFP plasmid DNA into chimeric VLPs was performed based on the VLP disassembly/reassembly method described previously^22^. Briefly, purified chimeric VLPs were treated with 1 mM ethylene glycol tetraacetic acid (EGTA) in the presence of 20 mM dithiothreitol (DTT) and further subjected to ultracentrifugation (200,000×*g*, 4 °C, 2 h). Approximately 5 µg of plasmid DNA was added into the disassembled VLPs overnight and CaCl_2_ was slowly added to the mixture to reach a final concentration of 5 mM. The mixture was then subjected to ultracentrifugation as the condition above.

Loaded MrNV-VLPs containing 1 µg pEGFP plasmid (as determined by A260) in culture medium were added to the SW480 and SW620 cell layers and allowed to incubate for 48 h. Cells incubated with unloaded VLPs or purified plasmids served as negative control and cells transfected with 1 µg pEGFP using Jetprime transfection reagent (Polyplus-transfection^®^ SA, NY) served as positive control. The delivery of the plasmid by the VLPs to SW480 (EGFR-positive) or SW620 (EGFR-negative) cells was assessed based on the expression of EGFP protein in the cell cytoplasm which was visualized by an Olympus FV10i confocal microscope as well as quantified by flow cytometric analysis.

To test the specificity of EGFR-mediated cargo delivery of chimeric VLPs using EGFR silencing method, SW480 cells were transfected with either EGFR siRNA as described in “Binding and internalization of chimeric VLPs in SW480 cells”. Two days after siRNA transfection, the delivery of encapsulated EGFP DNA by chimeric GE11-MrNV VLPs (N, MrNV, R-MrNV and E-MrNV) was assessed based on the expression of EGFP protein after 48 h of incubation using fluorescence microscope and flow cytometric analysis as described below.

### Indirect immunofluorescence confocal microscopy and flow cytometry

For indirect immunofluorescence (IIF), aldehyde-fixed cells were treated with 30 mM glycine in PBS and washed twice with PBST. Non-specific antibody staining was blocked with 4% BSA in PBST prior to incubation with a monoclonal mouse anti-MrNV antibody (1:500). The cells were washed and incubated with Alexa 488-conjugated goat anti-mouse antibody at a dilution of 1:1000 (Invitrogen). After washing, cells were counterstained with 4′, 6-diamidino-2-phenylindole dihydrochloride (DAPI) for nuclear staining and mounted with 50% glycerine in PBS (1:1, v/v). The stained cells were observed under an Olympus FV1000 confocal microscope (Olympus, Tokyo, Japan) using the krypton and argon laser excitation lines and either 350 nm (blue) or 520 nm (green) band-pass emission filter. All images were acquired under a Kalman line-by-line scanning mode to prevent any cross talk of the fluorescence emission.

For flow cytometry, SW480, SW620 and MCF7 cells treated with N-MrNV-VLP and chimeric GE11-MrNV-VLPs at different time points were trypsinized and fixed in 4% prechilled paraformaldehyde in PBS. Cells were washed with PBS and permeabilized (4 °C, 5 min) in 0.3% saponin in PBS. After blocking, cell were incubated with anti-MrNV and the corresponding Alexa 488 conjugated secondary antibodies under similar conditions described above. Data of approximately 1 × 10^4^ cells were collected with a BD FACSCanto™ flow cytometer (BD Biosences, San Jose, CA). Along with the green fluorescent signal of Alexa 488, the forward and side scatter parameters (FSC and SSC) were also recorded for differentiating the single cells from the surrounding debris and doublets. The data were further analyzed by FACSDiva™ Software (BD Biosciences, San Jose, CA). Plots represented mean ± SEM of normalized data. When the binding and internalization ability between GE11-MrNV VLPs vs. N-MrNV-VLPs were compared, the data were presented as the percentage of GE11-MrNV^+^ cells normalized with respect to the percentage of N-MrNV^+^ cells (%GE11-MrNV^+^/%N-MrNV^+^) × 100%. For the EGFR siRNA treatment to inhibit the EGFR-specific binding of each type of MrNV VLPs, the data were presented as the median fluorescence intensity (MFI) of EGFR-siRNA-treated cells normalized with respect to the MFI of Luc-siRNA-treated cells (control), (MFI siEGFR/MFI siLUC) × 100%.

## Results

### Homology-based structural model analyses of the chimeric GE11-MrNV-VLPs

Figure 1 showed the 3D homology models of 3 chimeric GE11-MrNV-VLPs: R-MrNV, I-MrNV, and E-MrNV, using N-MrNV structure (PDB# 6H2B) as a template.The deduced 3D models suggested that the placement of GE11 peptide did not greatly affect the 4-pillar structure of P-domain or overall VLP structure. In R-MrNV, the GE11 peptide completely replaced the amino acids at positions 352–363 of MrNV-VLP without interfering the main structure of the original P-domain (Fig. 1B). Structure of I-MrNV still revealed the 4-pillar structure upon the insertion of the GE11 peptide into the pillar 4 (between V354 and D355) of P-domain. The peptide appeared to protrude outward from this pillar and enlarge it to a certain extent, while the rest of the P-domain appeared intact. In E-MrNV, the main 4 pillar structure of P-domain remained intact, while the extending peptide was located at the side of the P-domain adjacent to that of pillar 4.

### Purified chimeric GE11-MrNV-VLPs retained their VLP structure

**Figure 2:**
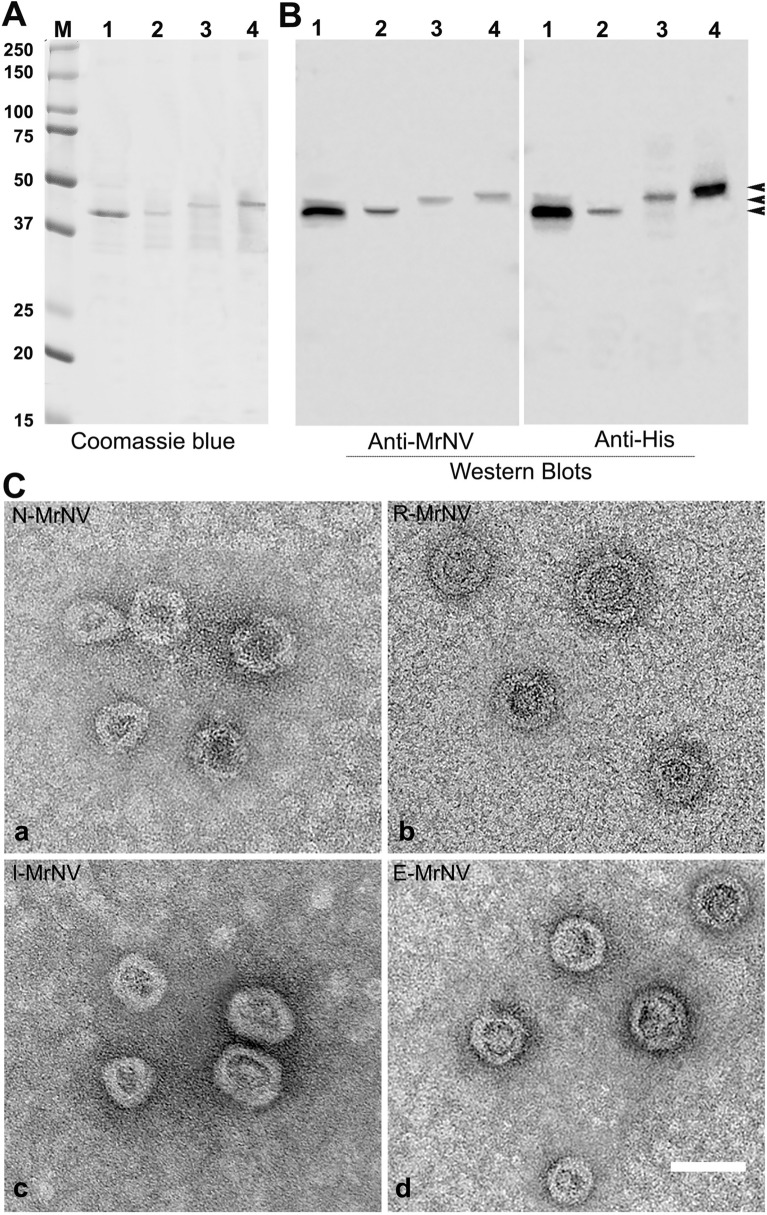
Protein profiles of chimeric GE11- MrNV-VLPs from SDS-PAGE and Coomassie blue staining (**A**) and their verification with Western blotting using anti-MrNV antibody or anti-His antibody (**B**). Lanes 1–4 represent N-MrNV, R-MrNV, I-MrNV, and E-MrNV, respectively. Electron micrographs of chimeric MrNV-VLP structures demonstrated by negative TEM staining are shown in panel (**C**). Bar = 50 nm.

All 3 types of chimeric GE11-MrNV capsid proteins produced in *E. coli* yielded the amount of purified proteins of about 2 mg/g of total proteins with the purity of about 90–95% in all samples. Protein profilings by a Coomassie blue staining of recombinant GE11-MrNV capsid proteins revealed a single major protein band with a molecular mass of about 42.5 kDa (for N-MrNV and R-MrNV), 43.0 kDa (for I-MrNV), and 43.5 kDa (for E-MrNV) (Fig. 2A). The proteins could be detected with both monoclonal anti-MrNV and anti-His antibodies (Fig. 2B) as a single band that had at the molecular weights corresponding to their Coomassie staining profiles.

In order to confirm the ability of these recombinant chimeric capsid proteins to undergo self-assembly to form VLP structure, all chimeric capsid protein samples were plated on formvar-coated grid, negatively stained and viewed under TEM microscope. The results in Fig. 2C indicated that all 3 chimeric capsid proteins formed a symmetrical mulberry-like VLP structure similar to that of the original MrNV-VLP that has been reported previously^22^. In addition, the size of all VLP particles generated were in the average range of 27–30 nm without any notable size alteration or apparent morphological changes from the original MrNV-VLP. These data verified that placement of GE11 short peptide in any three modes of chimerization did not affect to VLP structural formation.

### GE11-modified MrNV-VLPs have an enhanced binding to colorectal cancer cells

**Figure 3:**
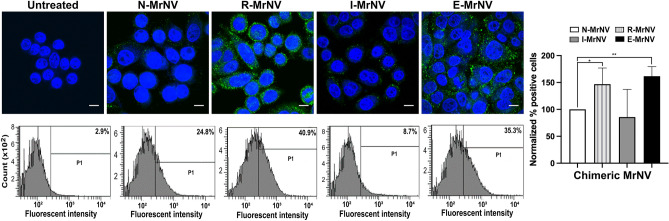
Binding of chimeric MrNV-VLPs to SW480 colorectal cancer cells. SW480 cells were incubated with all types of chimeric MrNV-VLPs and were stained with anti-MrNV and the corresponding secondary antibody conjugated with Alexa 488 (green) and either viewed by confocal microscopy (upper rows, bars = 10 μm) or analyzed by FACSCanto flow cytometer and FACSDiva software version 4.1.1 (https://bd-facsdiva.software) (lower rows). The percent MrNV positive cells and their statistical analysis were shown in the right-most panels (mean ± SEM, n = 3, *p < 0.05, Student’s *t* test).

Having created the 3 chimeric VLPs with sound assembly, we further investigated their ability to bind to the EGFR-positive colorectal cells (SW480). Following the incubation of the cells with different chimeric VLPs at 4 °C, the binding of R-MrNV and E-MrNV particles could be noted in the cells as an intense, punctate fluorescent staining by anti-MrNV antibody (Fig. 3, upper panels). It was apparent that the immunoreactivity of the chimeric R-MrNV and E-MrNV VLPs was more than those of N- MrNV-VLP and I-MrNV-VLP (Fig. 3, upper panels). The chimeric VLP binding was then quantified using flow cytometric analysis (Fig. 3, lower and right panels). The results clearly showed that R-MrNV and E-MrNV bound to the SW480 cells at about 1.4 and 1.6 fold (Fig. 3, right panel) more efficiently than N-MrNV VLPs. Notably, the binding efficiency of the I-MrNV did not improve, when compared to the N-MrNV. This suggests that the GE11 peptide that protruded from the P-domain structure failed to contribute to the binding ability to the cells. It is also probable that this protruded GE11 peptide may impair the general cellular binding of the MrNV in some circumstances. Overall these results showed that replacing a portion of the P-domain with GE11 peptide (as in R-MrNV) or adding the GE11 as an extension to the P-domain (as in E-MrNV) can enhance the ability of MrNV VLPs to bind to cancer cells.

### EGFR-dependent cellular binding of chimeric GE11-MrNV VLPS

**Figure 4:**
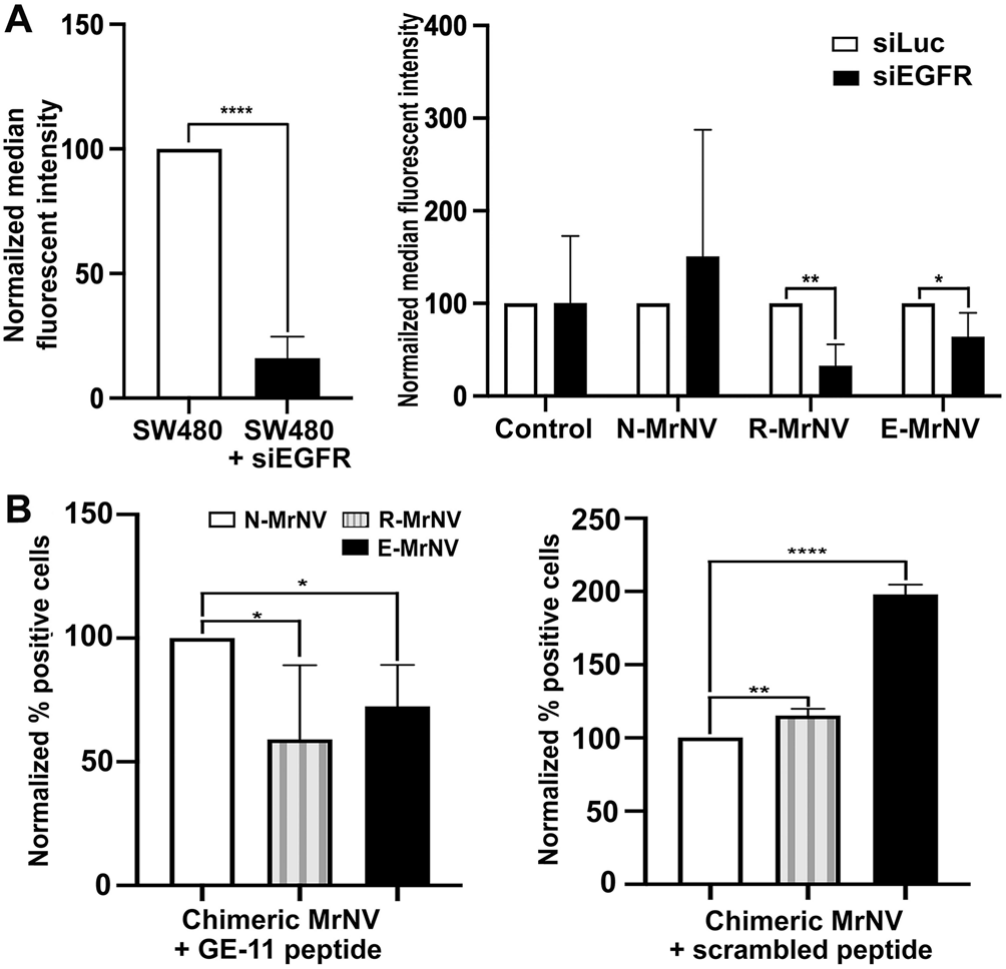
Inhibition of chimeric VLP binding through a silencing of EGFR by siRNA or interference with GE-11 peptide. SW480 cells were treated with siRNA against EGFR (**A**) or with GE11 peptide (**B**) prior to incubation with chimeric Mr-NV-VLPs and quantified the binding by flow cytometric analyses. Controls were SW480 cells treated with siRNA against luciferase (**A**, siLuc) or scramble peptide (**B**). * and ** denote statistical differences at *p* < 0.05 and 0.01, respectively (Student’s *t* test, n = 3, graphs represent mean ± SEM).

Since R- and E-MrNV showed superior binding ability to SW480, we further tested their EGFR-specific binding by knocking down EGFR expression by siRNA. The level of EGFR expression was blocked by about 80% in the SW480 upon treating with siEGFR (Fig. 4A, left panel). The EGFR silent cells showed a significantly lower binding towards R-MrNV (*p* < 0.01) and E-MrNV (*p* < 0.05). It should be noted that the binding of R-MrNV was more greatly affected (about 70%) than that of E-MrNV (about 45%) (Fig. 4A, right panel), suggesting the different degrees of involvement of EGFR in the binding of the two different chimeric GE11- MrNV VLPs.

We additionally performed an interference of VLP binding to SW480 cells with the well-known EGFR ligand, GE11 peptide. Both IIF and flow cytometric results clearly demonstrated that the excess GE11 peptide significantly inhibited (*p* < 0.05) the binding of the chimeric GE11-MrNV VLPs to the cell surface (Fig. 4B, left panel). No apparent binding inhibition was observed in cells treated with the scrambled peptide (control; right panel), namely, they showed similar binding ability as those of non-peptide treated cells (compared to Fig. 3).

Both siEGFR and GE11 peptide competition assays strongly indicate that modifying MrNV VLPs with GE11 peptide enhances the cancer cell-binding of the VLPS in an EGFR-specific manner. This notion is also supported by our observation that all types of chimeric GE11-MrNV VLPs showed little binding to EGFR-negative MCF7 breast cancer cell line and no significant increase in the binding was observed when compared to the N-MrNV (Supplementary Fig. S1).

### Chimeric MrNV-VLPs were efficiently internalized into SW480 cells

**Figure 5:**
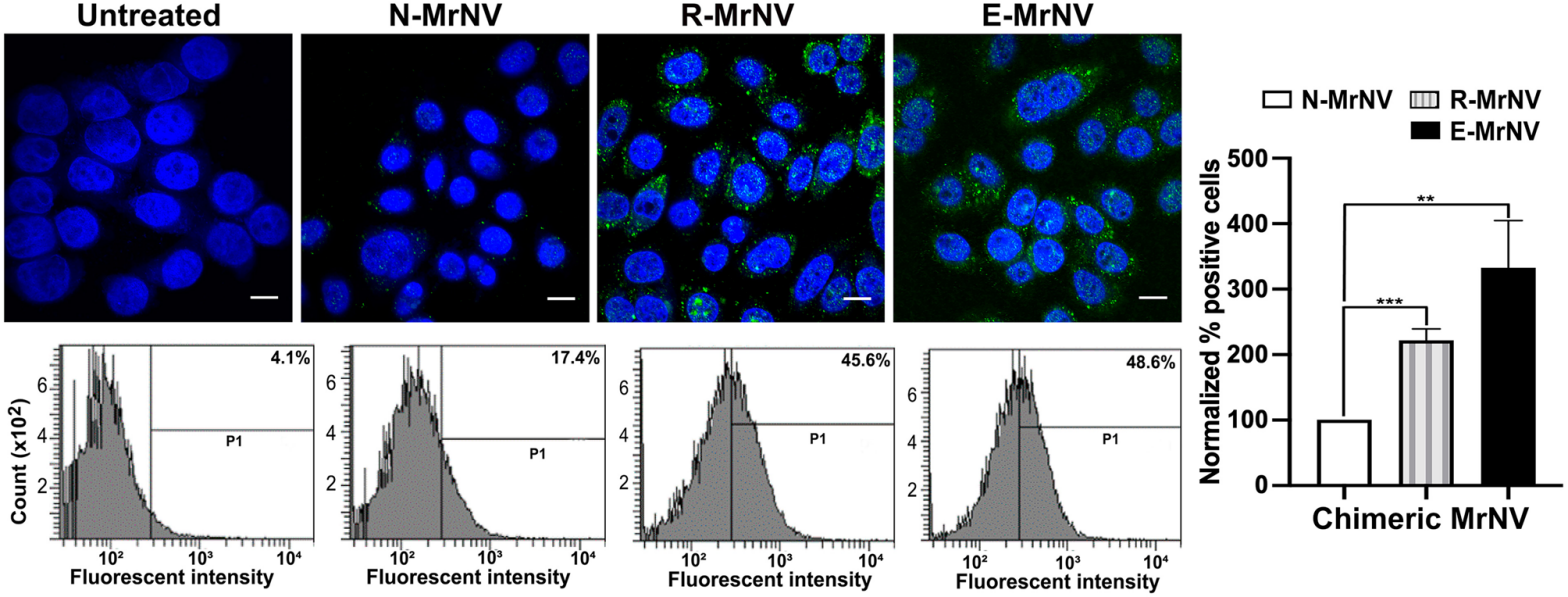
Internalization of chimeric MrNV-VLPs to SW480 cells. SW480 cells were processed similarly as binding assay with all types of MrNV-VLPs followed by an additional 30 min incubation after cell washing and further analyzed with confocal microscopy (upper row, bars = 10 μm) and flow cytometry (lower row). The normalized percent positive cells and their statistical analysis were shown in the right-most panels (mean ± SEM, n = 3, *p < 0.05, Student’s *t* test).

Ligand-induced EGFR internalization is a well-recognized process^30^. After confirming the EGFR-specific binding of the chimeric MrNV VLPs, we further investigated the EGFR-dependent internalization of the chimeric VLPs. The IIF results demonstrated that after 30 min of synchronized internalization, the dots of immunoreactivity were clearly identified at the periphery of the cytoplasm especially underneath the cell membranes and supranuclear region in cells treated with E-MrNV and R-MrNV, suggesting the successful cellular binding and internalization of these chimeric MrNV (Fig. 5, upper panel). The 3D confocal images and their multi-axial sections clearly demonstrated internalization of VLP particles within the cytoplasm of SW480 cells (Supplementary Fig. S2). In line with the less efficient binding N-MrNV VLPs to the SW480 cells, significantly less immunoreactivity was observed for these VLPs. The flow cytometric results quantitatively confirmed this observation (Fig. 5, lower panels). These results confirmed that the R-MrNV and E-MrNV, which could bind more effciently to the cells in the EGFR-specific manner than the N-MrNV were internalized into the cells more efficiently (up to two- to threefold higher) when compared to the N-MrNV. This enhanced uptake was likely attributed to the EGFR-dependent internalization.

### The chimeric MrNV GE11-VLPs specifically delivered encapsulated cargo to the targeted EGFR-positive cancer cells

The ability of chimeric MrNV VLPs to encapsulate EGFP plasmid DNA at about 40–50% was as expected^22,23^. The ultrastructures of these chimeric VLPs during their disassembly and re-assembly process to encapsulate EGFP plasmid (as revealed by TEM negative staining, Supplementary Fig. S3) was similar to the structure of N-MrNV reported previously^22^. To further confirm that EGFP plasmid was loaded into the VLP cavity and not only adhering on the cell surface, the loaded VLPs were treated with 1 U of DNaseI for 1 h to digest any exteriorly bound DNA. The results revealed that the band of intact plasmid at 5.5 kb was notable, suggesting the successful encapsulation and protection of the plasmid DNA inside the VLPs (Supplementary Fig. S3).

**Figure 6:**
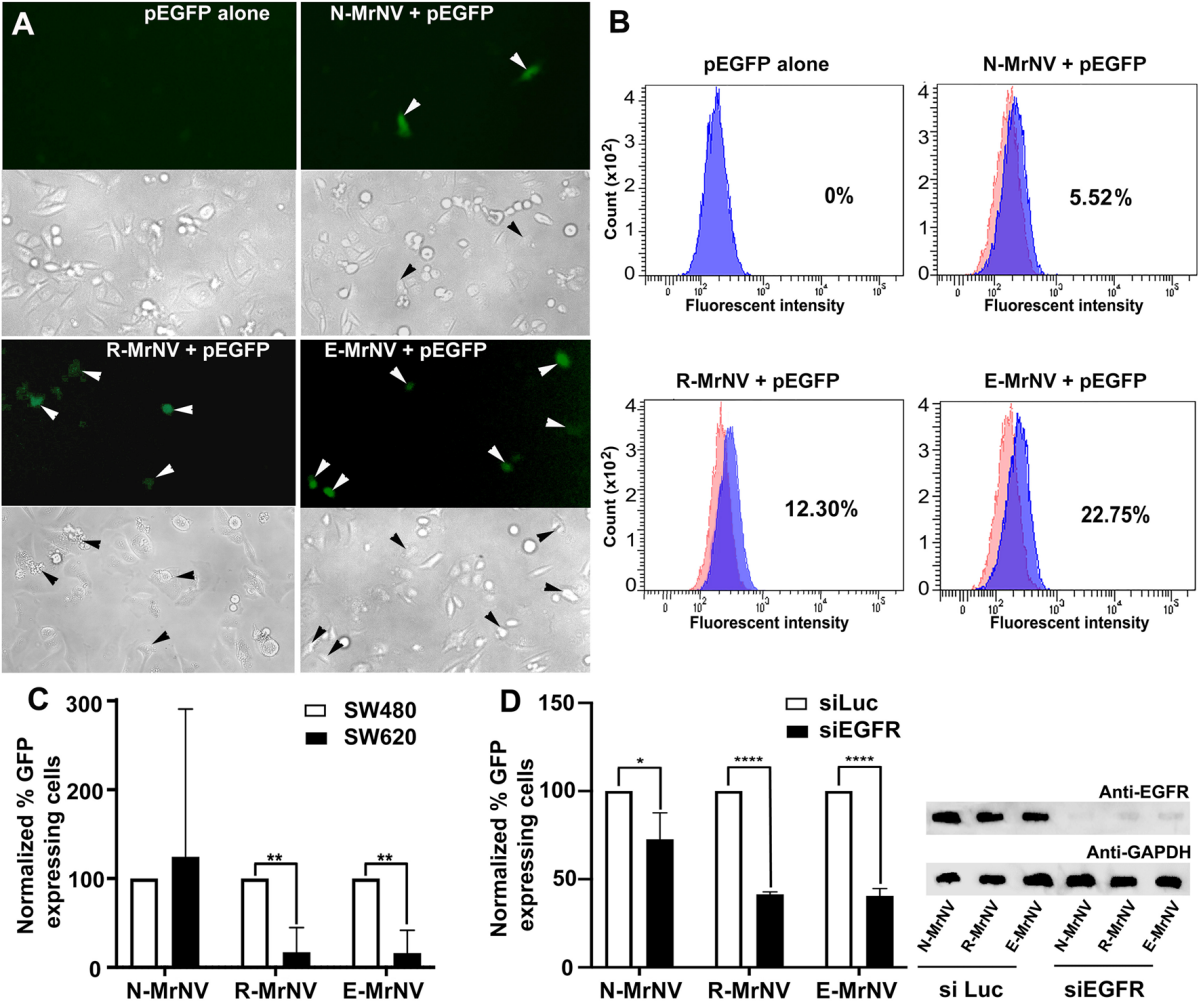
Chimeric MrNV-VLPs delivered encapsulated EGFP plasmid specifically to EGFR-positive cells. Both EGFR-positive colorectal cancer cells (SW480) and EGFR-negative cells (SW620) were incubated with MrNV VLPs carrying its cargo, encapsulated EGFP plasmid, and were left further to allow GFP protein expression for 48 h. The cells were then either visualized by confocal microscopy (panel **A**) or flow cytometry (panel **B**). Flow cytometric analyses of GFP expression in SW480 and SW620 cells (panel **C**) or those treated with siRNA against EGFR or luciferase (siLuc, control) in SW480 cells (panel **D**; right, typical EGFR knockdown level shown by immunoblotting).

After 48 h incubation with VLPs loaded with the EGFP plasmid, an EGFP fluorescent signal in SW480 cells treated with both R-MrNV and E-MrNV could be clearly visualized (Fig. 6A). Apparently less EGFP positive cells were detected when cells were treated with EGFP-loaded N-MrNV. No fluorescent signal was observed in cells incubated with only plasmid DNA. Flow cytometric analysis confirmed the EGFP plasmid delivery to SW480 cells by R-MrNV and E-MrNV VLPs with a ~ 2.5 to 4-fold increase in EGFP positive cells compared to the N-MrNV VLPs (Fig. 6B). When the SW620 cells, which are colorectal cancer cells derived from the same patient as the SW480 but lack EGFR expression, were treated with EGFP-encapsulated R-MrNV and E-MrNV VLPs, the significantly lower percentages of EGFP positive cells in both chimeric VLPs (*p* < 0.01) were noted when compared to the percentages of EGFP-positive SW480 cells (Fig. 6) This suggested the lack of EGFR-specific enhancement of cargo delivery in these cells, as expected for EGFR-negative cells.

We further performed EGFR suppression using siRNA against EGFR (siEGFR) on SW480 cells in order to determine the dependence of the cargo delivery by the VLPs on EGFR expression. Generally, cells treated with control siRNA (siLuc) always showed a higher plasmid delivery and thus higher levels of cells expressing EGFP compared to those treated with siEGFR. Notably, for the EGFR-knockdown cells treated with EGFP-loaded R-MrNV and E-MrNV, the levels EGFP-positive cells reduced markedly (*p* < 0.001), with 60% less EGFP- expressing cells than the siLuc control counterpart (Fig. 6D). These results clearly demonstrated that the chimeric R-MrNV and E-MrNV VLPs delivered the cargo with high specificity to EGFR-positive cancer cells.

## Discussion

Application of VLPs becomes a well-established platform that provides the solution for specifically targeted drug delivery because of their abilities to encapsulate several types of cargoes after systemic functionalization of VLP for enhancing receptor recognition. In addition, through genetic engineering of the available capsid sequences, several modes of capsid alterations either via insertion, deletion or substitution of ligands on the specific site(s) of the capsid proteins are rather practical. Such modifications apparently improve biological and physiological properties of VLPs^31–34^. In this regard, we have successfully modified hepatitis E (HEV)-VLP by inserting p18 peptide (derived from the V3 loop of HIV-1 gp120) into the P-domain sequence of HEV capsid protein which alters the surface property of chimeric VLPs and allows them to escape from anti-HEV reactivity^35^. Similarly, engineering of Qβ-VLP by inserting human epidermal growth factor (EGF) into its exterior improves the interacting property of this chimeric VLP in inducing autophosphorylation of EGFR and enhancing apoptosis of A431 cells^13^. In this study, placement of GE11 peptide into the pillar structure of MrNV-VLP apparently altered VLP’s interaction property towards the human cancer cell line, which otherwise its interaction with human cells would be negligible (Figs. 4, 5 and 6). Higher specificity of chimeric VLP targeting is likely derived from an interaction of GE11 peptide with its known targeting receptor, EGFR, on the cell surface^27^. One excellent example to show GE11 specificity is the genetic fusion of GE11 into adenovirus 5 (Ad5) virus particle at the AB loop of the viral fiber knob (so called KO1 mutation) which significantly decreases binding of Ad5 towards the recipient cells while reversely enhances the internalization of the chimeric Ad5 into EGFR positive cell lines^36^. The other example of chemically modified nodavirus (Flockhouse virus or FHV, structurally homolog to MrNV) with tumour homing peptide has shown recently to successfully deliver hydrophobic drugs into EGFR-positive breast cancer cells, a closely-resembled example to MrNV reported herein^37^.

Apart from GE11 specificity, our results in this study clearly showed the differential binding ability of chimeric GE11 + MrNV-VLP variants towards EGFR positive SW480 cells. Notably, R-MrNV-VLP and E-MrNV-VLP have a far superior binding ability to that of I-MrNV and N-MrNV-VLPs (Fig. 1). We believe that the stoichiometry and conformation of GE11 upon its placement into the MrNV-VLPs are among the important factors that determine the specific binding ability of the chimeric VLP. The most favorable site of ligand or short peptide insertion into the outer surfaces of many types of VLPs is located at P-domain where the native virions present their interaction ligand towards host cell receptors^13,24,29,35,38^. In case of MrNV, previous studies have shown that the P domain localized at the C-terminus of MrNV capsid plays an important role in binding and internalization in the host cells^24,29,39^. The atomic structure of MrNV resolved from cryo-EM indicated that the exposing surface of the P domain exhibits 4 loop- or pillar-structure located at amino acids 268–355 that represent potential insertions for targeting peptide^24^. In this study, placement of GE11 peptide at the 4th pillar (positioned 352–363 or R-MrNV) showed a high efficiency of chimeric MrNV-VLPs to interact with EGFR receptor on the cancer cell surface (Fig. 4). This is not unexpected, since our previous results have indicated that truncation of 20 terminal amino acids (positioned 352–371) at MrNV’s C-terminus by chymotrypsin digestion results in a strong adverse effect on VLP binding and internalization as compared to its normal MrNV-VLP counterpart^39^. In addition, the position where GE-11 peptide is located on the surface of R-MrNV should possess the closest structural conformation similar to the original peptide orientation on the P-domain of MrNV, which presumes to offer the best presentation of the ligand towards its receptor as with the virion interaction to the host cells. It is worth noting that the extension of the GE-11 peptide (E-MrNV-VLP variant) also offered an efficient binding and internalization of chimeric VLPs into the cancer cells. This may be explained by the non-disturbance of the P-domain’s pillar structure by the addition of GE11 peptide, while the overhanging of the short peptide with the linker would allow more flexibility to freely display ligand towards the exposing EGFR receptor. This is in agreement with the previous studies of MrNV where they were engineered with the extended M2e (matrix 2 protein) or hepatitis B surface antigen (HBsAg) on the C-terminus of MrNV capsid and exert the specific property in inducing immunity in vivo^25,26^.

Here, we also launched our effort to encapsulate EGFP plasmid into the cavity of chimeric VLPs with similar encapsulating efficiency as reported earlier in normal MrNV^22^. Loading a considerable amount of genetic materials into VLP’s cavity has long been performed without any interior modification of the VLP^22,40^. This is simply due to the possession of many positively charged amino acids at the *N*-terminus of capsid sequence which is finally folded into the cavity of virion quaternary structure of many non-enveloped virus species^24,41–43^. This case also holds true for MrNV where the 1–30 amino acids at N-terminus (also called N-ARM) is enriched with Arg and Lys amino acids and plays a role in binding to negatively charged RNA. This N-ARM domain regulates both length and conformation of ssRNA while interacting with viral capsid interior^24^. Although this inherent property of MrNV-VLP has already been advantageous for encapsulating nucleotide-based materials into the VLP’s cavity, however, its cargo loading capability can still be maximized to improve the efficiency of the therapeutic compound on the site of the target. This is particular for the case of cancer therapy or DNA vaccination where the local concentration of an active compound that is delivered into target cells is of utmost importance. In this perspective, the future avenue is to modify the interior of chimeric MrNV-VLP with the aim to maximize the encapsulation of active cancer therapeutic compounds. Conclusively, this study demonstrated the utility of chimeric MrNV nanocontainers rationally designed for specific delivery of cargoes, such as DNA and anticancer drugs, to targeted cancer cells. Further development of nanocontainers based on this study benefits the quest for improved targeted therapies.

## Supplementary Information

Supplementary Figures.
