# Supplementary material for: Chimeric virus-like particles (VLPs) designed from shrimp nodavirus (MrNV) capsid protein specifically target EGFR-positive human colorectal cancer cells
Source: Sci Rep. 2021 Aug 16;11:16579. doi: 10.1038/s41598-021-95891-x (PMC8367941; doi:10.1038/s41598-021-95891-x)
Supplement: Supplementary file 1 — Supplementary Figures. [file 41598_2021_95891_MOESM1_ESM.pdf]

Supplementary Figures for Manuscript Entitled:

Chimeric virus-like particles (VLPs) designed from shrimp nodavirus (MrNV) capsid protein specifically target EGFR-positive human colorectal cancer cells

Khwanthana Grataitong, Sébastien Huault, Charoonroj Chotwiwatthanakun, Pitchanee Jariyapong, Orawan Thongsum, Chidchanok Chawiwithaya, Krittalak Chakrabandhu, Anne-Odile Hueber, Wattana Weerachatyanukul

Supplementary Figure S1

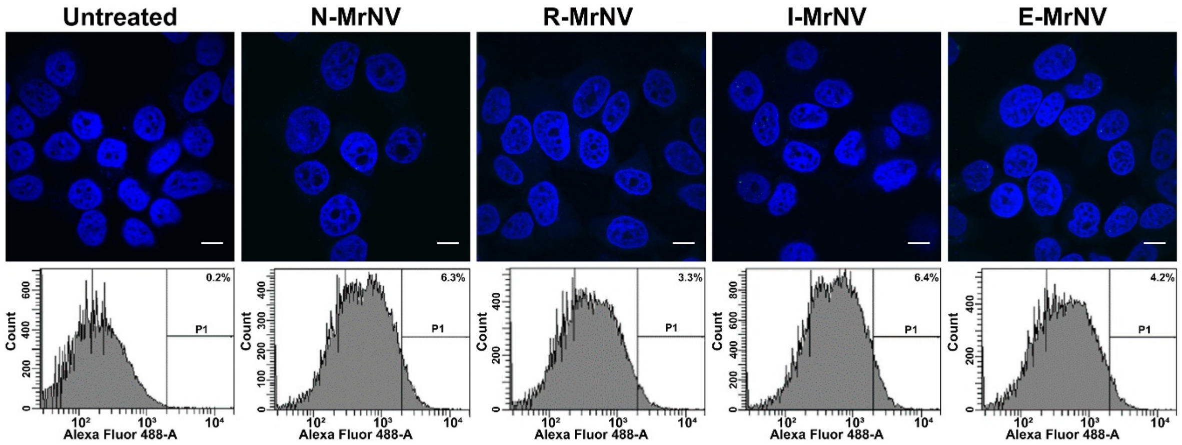

Figure S1 Binding of chimeric MrNV-VLPs to EGFR-negative MCF7 breast cancer cells. MCF7 cells were subjected to the binding assay with all types of chimeric MrNV-VLPs and were stained with anti-MrNV and the corresponding secondary antibody conjugated with Alexa 488 (green) and either viewed by confocal microscopy (upper rows, bars = 10 μm) or analyzed by flow cytometry (lower rows).

Supplementary Figure S2

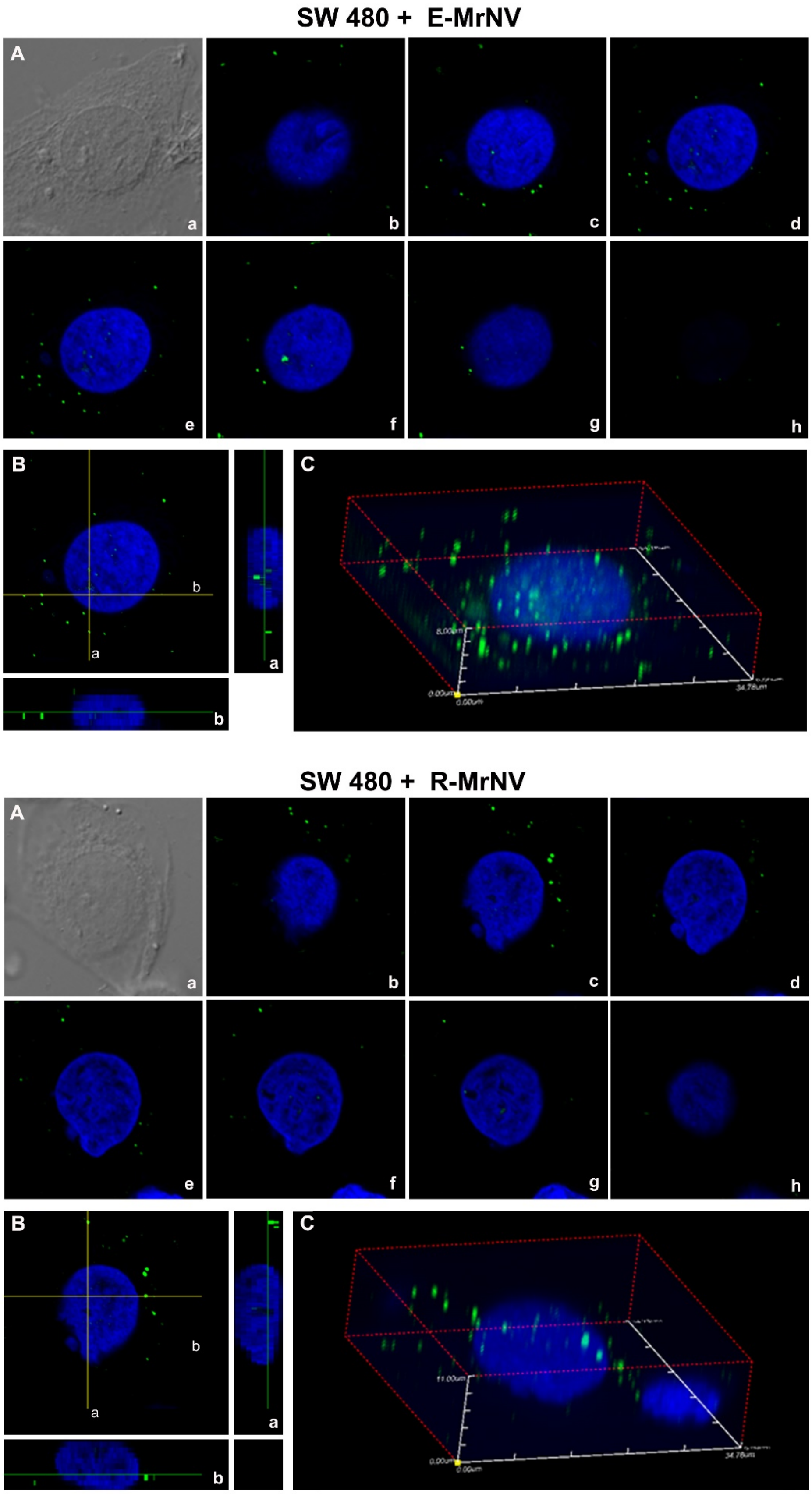

Figure S2 Confocal microscopic images of chimeric MrNV internalization into SW480 cells. The cells were incubated either with E-MrNV (upper panels) and R-MrNV (lower panels) and stained with anti-MrNV antibody followed by its corresponding secondary antibody conjugated with Alexa 488 (green) and counterstained by DAPI nuclear staining (blue). The selected Z-stack serial sections are shown in A, while the longitudinal and transverse sections as well as its three-dimensional reconstruction are shown in panels B and C, respectively.

Supplementary Figure S3

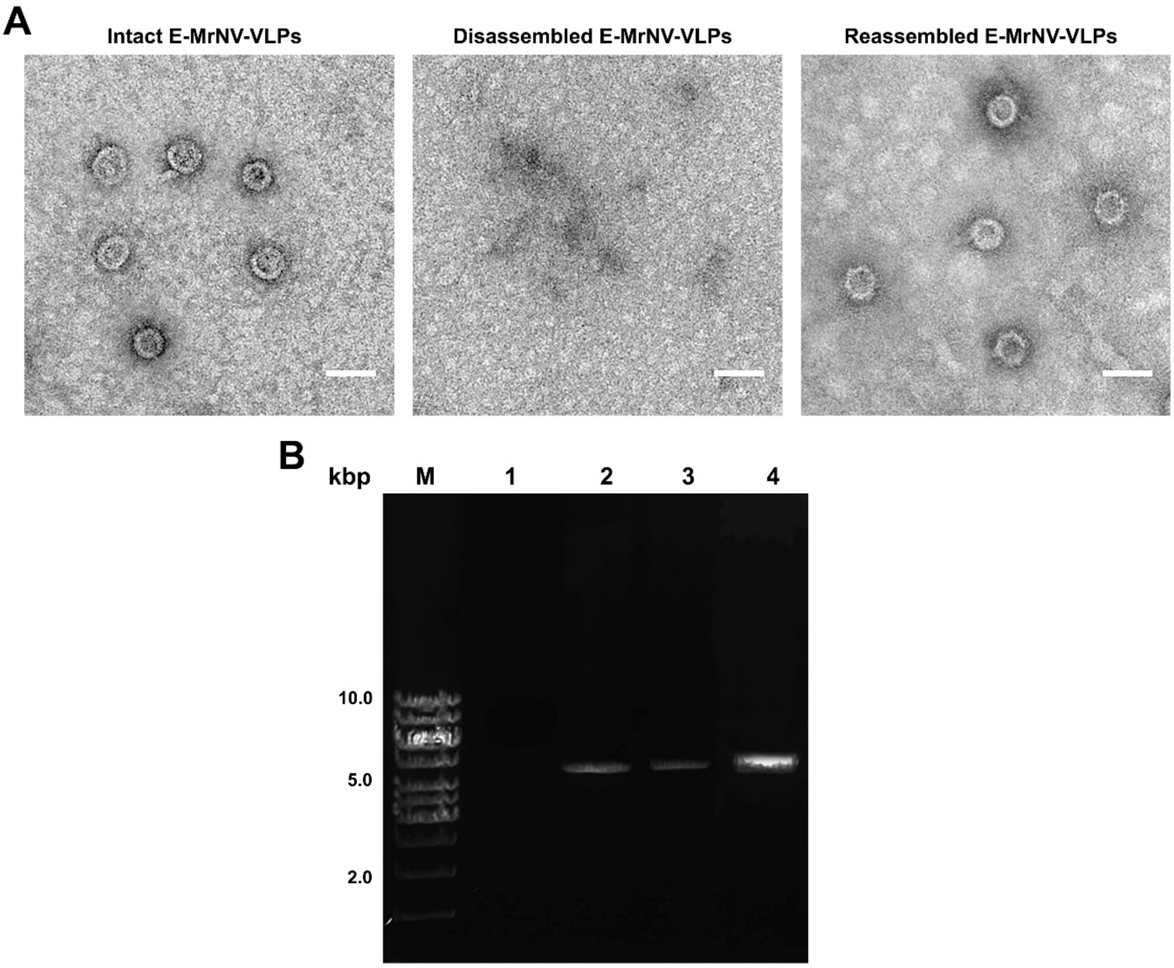

Figure S3 Representative TEM images demonstrating disassembly and reassembly of E-MrNV-VLPs to encapsulate EGFP plasmid into VLP's cavity. A: transmission electron micrographs of intact (left), disassembled (middle), and reassembled VLPs (right). B: gel electrophoresis of empty E-MrNV (lane1), E-MrNV+EGFP plasmid (lane2), E-MrNV+EGFP plasmid+DNaseI (lane3), and free EGFP plasmid shown as a single band at 5.5 kbp (lane4).

Raw data of Figure 2 (Coomassie staining and Western blots)

Fig. 2A

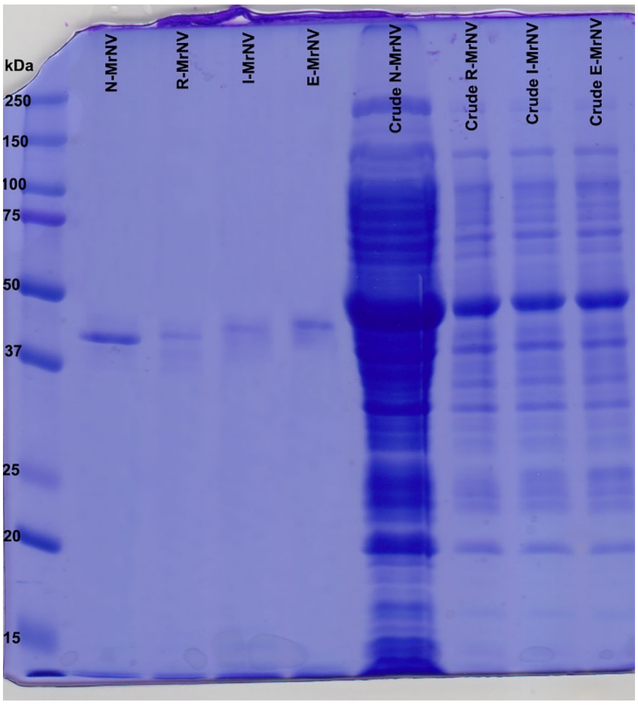

Fig. 2B (Anti-MrNV, left panel)

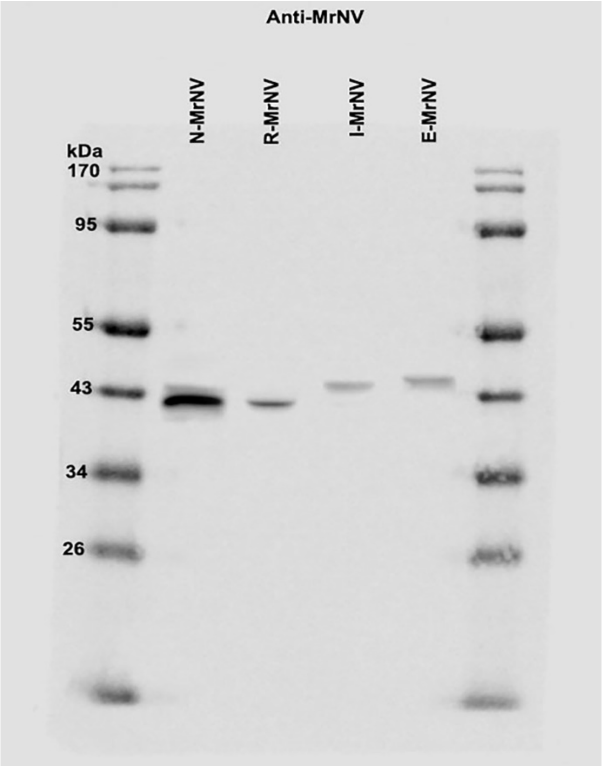

Fig. 2B (Anti-His, right panel)

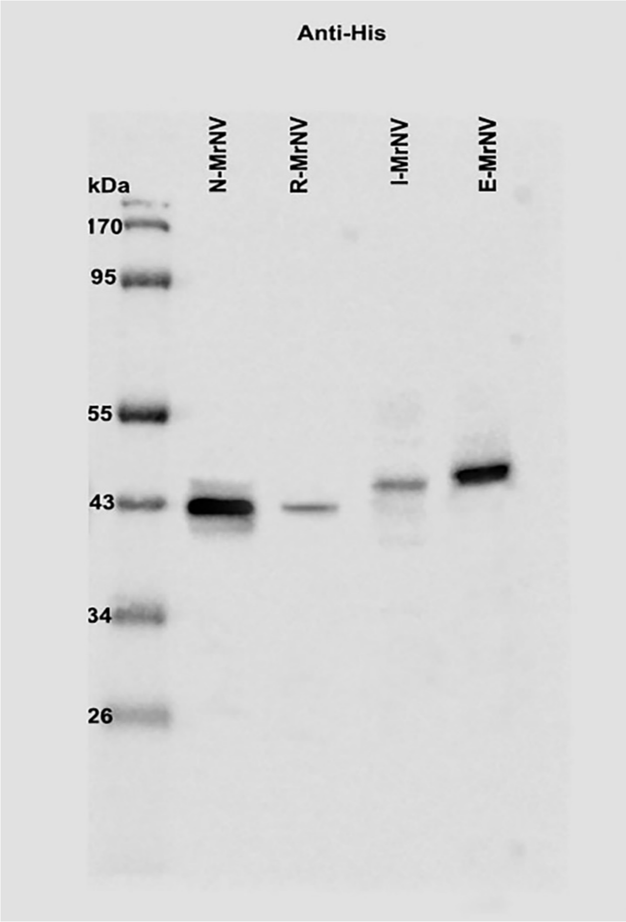

(**Figure legend of this supplementary information is similar to Figure 2**)

Raw data of Figure 6D (Slot-Blot analysis)

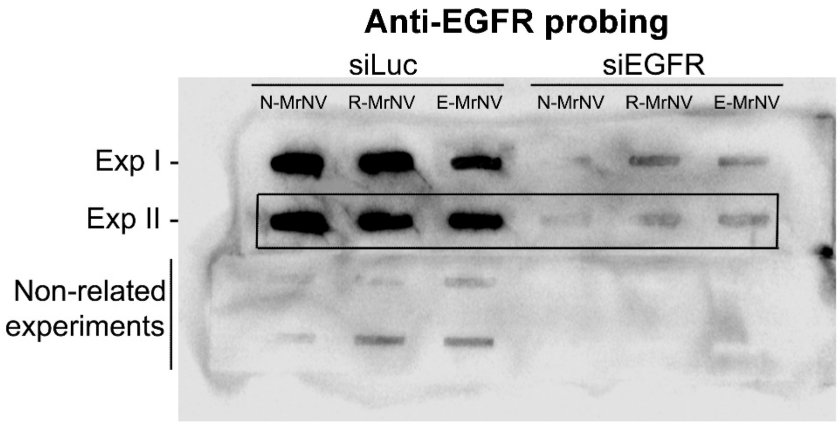

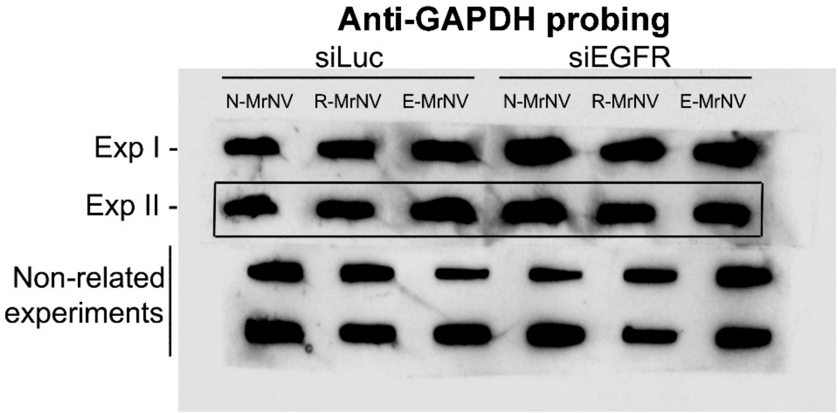

Figure 6D (right panel) Slot blot analysis showing down regulation of EGFR expression after siEGFR treatment. SW480 cells were subjected to either siRNA against EGFR (right) or siLuc (left) treatments and further exposed to chimeric MrNV (N-, R-, and E-MrNV) incubation. Cell lysates were transferred to PVDF membrane and probed with either anti-EGFR (upper panel) or anti-GAPDH (lower panel) and the corresponding HRP-conjugated secondary antibody. The solid boxed areas in the membrane corresponded with the blots shown in Fig. 6D (derived from duplicated experiments, Exp I and Exp II). The whole strips of cut PVDF membranes (including the blots of non-related experments which were exposed at the same time) where the edges of the membranes could be visualized are shown.
